# Metabolic Pattern of Systemic Sclerosis: Association of Changes in Plasma Concentrations of Amino Acid-Related Compounds With Disease Presentation

**DOI:** 10.3389/fmolb.2020.585161

**Published:** 2020-10-15

**Authors:** Zaneta Smolenska, Magdalena Zabielska-Kaczorowska, Anna Wojteczek, Barbara Kutryb-Zajac, Zbigniew Zdrojewski

**Affiliations:** ^1^Department of Internal Medicine, Connective Tissue Diseases and Geriatrics, Medical University of Gdańsk, Gdańsk, Poland; ^2^Department of Human Physiology, Medical University of Gdańsk, Gdańsk, Poland; ^3^Department of Biochemistry, Medical University of Gdańsk, Gdańsk, Poland

**Keywords:** systemic sclerosis, amino acids, metabolome, endothelium, inflammation

## Abstract

**Objective:**

Amino acids (AA) and their derivatives play an integral role in the synthesis of structural and regulatory elements in human organisms and therefore pathologies such as systemic sclerosis that may alter the blood pattern of these compounds. This study aimed to evaluate changes in plasma concentrations of amino acid-related metabolites in systemic sclerosis in a search for potential biomarkers and mechanisms of the disease.

**Methods:**

Plasma samples from 42 patients diagnosed with systemic sclerosis (SSc) according to the 2013 American College of Rheumatology and European League Against Rheumatism ACR/EULAR classification criteria were compared to 27 matched healthy controls. Liquid chromatography/mass spectrometry was applied for the analysis of 36 amino acid-related metabolites.

**Results:**

The analysis of plasma AA metabolite patterns revealed the number of changes including an increase (20%) in concentrations of NO synthase (NOS) inhibitor asymmetric dimethylarginine (ADMA) in SSc vs. healthy subjects. Furthermore, SSc patients had higher glutamine, proline, betaine, 1-methylhistidine, and methylnicotinamide levels, while the concentration of tryptophan was lower. The specific metabolic pattern was identified for several aspects of disease presentation. Most interestingly NOS inhibitor L-NAME was elevated in patients with diffuse systemic sclerosis or telangiectasia.

**Conclusions:**

These results provide further evidence for the involvement of endothelium-dependent pathways in the mechanisms and presentation of SSc. Endothelial dysfunction biomarkers may be useful in the assessment of presentation and prognosis in SSc.

## Introduction

Systemic sclerosis (SSc) is an autoimmune connective tissue disease characterized by fibrosis of the tissues, preceded by microvascular alterations and immune dysfunction (Zanatta et al., 2019). The proliferation of fibroblasts with the production of excessive extracellular matrix leads to the thickening of the skin and damage to internal organs. It is a highly heterogeneous disease with a wide variety of clinical presentations and is connected with increased morbidity and mortality (Barsotti et al., 2019). SSc can occur in two main cutaneous subsets: diffuse systemic sclerosis (dcSSc) and limited systemic sclerosis (lcSSc) (Young and Khanna, 2015). In the lcSSc subset, skin fibrosis is mainly restricted to distal extremities and the face, with slow accumulation of organ involvement, while Raynaud’s phenomenon typically occurs even years before any skin and visceral changes. By contrast, the dcSSc subset presents a more aggressive progression characterized by severe internal organ manifestations with skin thickening extended proximally to elbows and knees as well as the trunk. In the dcSSc subtype, anti-topoisomerase I autoantibodies (anti-Scl-70) are more frequent, while in the lcSSc subtype, anti-centromere autoantibodies are dominant features (Kranenburg et al., 2016).

In 2013, EULAR published new criteria for SSc, which can help early diagnose this illness (Van Den Hoogen et al., 2013). These include main clinical features as extending of skin involvement, finger changes as sclerodactyly, puffy fingers, digital ulcerations and pitting scars, telangiectasia, the presence of lungs complications (pulmonary hypertension and/or interstitial disease) but other manifestations as from GI tract, heart, arthritis with tendon inflammation, or calcinosis in the skin also occur commonly in SSc. Many studies have shown that dcSSc characterized by rapid progression of skin thickness has been associated with earlier onset of heart, lung involvement, and also with increased disease severity and mortality rates due to this organ involvement (Kumánovics et al., 2017). Predicting which organs are likely to be involved, and better understanding who is at the risk for potentially devastating complications is critical for appropriate patient counseling.

Metabolomic profiling including the analysis of plasma amino acid balance is a new, promising biochemical approach that can be applied for SSc screening, diagnosis, disease typing, and treatment monitoring, as well as may be promising for SSc biomarker discovery (Sandlers, 2020). Amino acids play a very integral role as metabolic intermediates and in the building block of proteins, while their plasma concentration highly depends on the organs’ functions and pathological conditions that alter human body metabolism (Bröer and Bröer, 2017).

This study aimed to evaluate the changes in plasma concentrations of amino acids and related metabolites in SSc depending on the clinical characteristics of patients and organ involvement regarding potential mechanisms of the disease. The additional goal was to investigate whether individual amino acids and their derivatives can be potential SSc biomarkers.

## Materials and Methods

### Patients

This study included 42 adult patients (35 women, 7 men, aged 59.9 ± 12.4; min–max: 28–78 years) diagnosed with SSc referred to the Outpatient Rheumatology Clinic of the Department of Internal Diseases, Connective Tissue Diseases and Geriatrics of the University Hospital in Gdańsk, Poland. The control group represented age and sex-matched individuals (*n* = 27) with no report of any pathology. All the investigation involving human subjects was performed in compliance with standards of the Declaration of Helsinki (1975/83). The study has been approved by the Bioethics Committee at the Medical University of Gdańsk, Poland. The diagnosis of SSc was established according to the 2013 American College of Rheumatology and European League Against Rheumatism ACR/EULAR classification criteria. The SSc patients were categorized into the limited (lcSSc, *n* = 21) or the diffuse cutaneous (dcSSc, *n* = 21) subsets. The modified Rodnan skin score was used for grading the cutaneous extension of sclerosis. Organ involvement was evaluated using double-contrast esophagography, high-resolution computed tomography (HRCT) scans, pulmonary function tests (PFTs), two-dimensional echocardiogram, X-ray, and ultrasonography of the musculoskeletal system. All patients were subjected to basic routine laboratory and serological tests. Further clinical features are summarized in Table 1.

**TABLE 1:** Demographic and clinical presentation of systemic sclerosis patients (*n* = 42).

Gender	85% F/15% M
Age (y)	59.9 ± 12.4 (28–78)
C-reactive protein (mg/L)	4.9 ± 4.3 (0.2–45.9)
Erythrocyte sedimentation rate (mm/h)	15.3 ± 12.3 (1.6–66.0)
Calcinosis	14%
Joint involvement	73%
Lung involvement	61%
Dysphagia	28%
Telangiectasia	65%
Scleroderma	49%
Upper GI tract involvement	45%
Heart involvement	17%

### The Analysis of Plasma Amino Acids and Nicotinamide Metabolites

The concentrations of plasma amino acids and related compounds were determined using liquid chromatography/mass spectrometry (LC/MS) according to our described procedure (Olkowicz et al., 2017) that expanded earlier use of the reversed-phase ion-paring approach for analysis of amino acids (Chaimbault et al., 1999). Briefly, an aliquot of plasma (0.05 mL) was spiked with internal standards and deproteinized with 0.1 mL of acetonitrile followed by maintenance on ice for 15 min. The tubes were then centrifuged at 4°C, 12,000 × *g* for 5 min. The supernatant was collected and freeze-dried. Samples were then dissolved in 0.1 mL of water and analyzed with the use of ion-pair high-performance liquid chromatography with mass detection. Chromatographic separation was performed using 2.5 μm Synergy Hydro-RP 50 × 2.0 mm column. The mobile phase was delivered at 0.2 mL/min in a gradient from 0 to 60% acetonitrile in 12 min. The mass detector (TSQ Vantage, Thermo, United States) with heated electrospray (HESI-2) ion source was operating in positive MS2 mode for the detection of amino acids. The electrospray cone voltage was set at 4.5 kV and a heated capillary temperature was 275°C. Sheath gas flow was set for 35 arbitrary units. Post column make-up flow of methanol with 0.05% formic acid at 0.2 mL/min was used to improve ionization efficiency. The identity of individual amino acids and their derivatives was confirmed by the similarity of molecular weights, fragmentation pattern, and chromatographic retention time. The procedure allowed interference-free quantitation of all analyzed compounds including challenging molecule pairs such as SDMA and ADMA that despite similar molecular weights produced different fragments in MS^2^ mode that were used for quantitation. Calibration was based on curve prepared in spiked plasma matrix as we have described in detail earlier (Olkowicz et al., 2017).

### Statistical Analyses

The significance of differences in the level of analyzed compounds between patients and controls or their association with pathology were evaluated by Student *t*-test or by Mann-Whitney’s *U*-test when variables distribution was not symmetrical (as confirmed by Shapiro-Wilks’ W test) or the groups of patients were much different from the sample size. All results are presented as mean ± S.D. with the minima and maxima observed. *p* < 0.05 was considered as a significant difference.

## Results

The comparison of amino acid (AA)-related metabolites concentration between systemic sclerosis patients and healthy control group is shown in Table 2. The patients have higher concentration of glutamine (12% increase over control = IOC), proline (17% IOC), 1-methylhistidine (39% IOC), betaine (23% IOC), methylnicotinamide (MNA, 35% IOC) and asymmetric dimethylarginine (ADMA, 19% IOC). The level of tryptophan was lower in the SSc group than in controls by 20%.

**TABLE 2:** The comparison of amino acid metabolite concentrations in systemic sclerosis patients with control healthy subjects.

	Control (*n* = 27)				Systemic sclerosis (*n* = 42)			
		
	Mean	S.D.	Min	Max	Mean	S.D.	Min	Max
Alanine	223.5	119.6	65.0	528.3	265.5	134.6	83.4	682.6
Asparagine	36.7	13.0	9.1	60.9	36.5	13.7	15.8	83.4
Aspartate	9.7	4.3	4.6	23.5	7.8	4.9	1.1	20.2
Arginine	67.8	22.1	17.8	113.6	76.7	26.8	38.2	167.7
Cystine	16.5	8.3	2.8	35.6	18.6	9.5	0.9	41.3
Glutamate	86.7	52.1	4.8	225.2	98.0	42.7	36.1	205.3
Glutamine	618.4	165.3	231.0	893.1	689.0*	122.3	457.2	971.1
Glycine	219.3	107.4	54.3	541.3	216.4	85.1	82.5	464.7
Histidine	88.6	21.6	31.7	120.5	93.0	27.7	43.6	166.0
Isoleucine	96.9	41.8	27.7	213.9	102.9	33.5	41.0	198.9
Leucine	129.5	36.3	28.3	190.6	131.3	31.9	81.2	233.7
Lysine	146.1	43.4	32.6	218.9	139.6	30.3	74.6	212.5
Methionine	33.3	12.4	4.7	55.5	29.8	9.0	13.5	52.7
Phenylalanine	69.0	24.2	20.1	140.9	74.8	30.1	42.0	207.5
Proline	152.5	47.3	23.7	247.1	178.8*	55.2	93.7	342.6
Serine	94.8	39.3	22.2	200.7	93.1	24.8	32.2	152.7
Threonine	141.8	52.6	34.5	238.5	141.2	41.2	77.1	282.6
Tryptophan	40.8	12.3	13.3	61.8	32.5**	9.6	11.5	66.6
Tyrosine	73.1	24.5	15.3	130.2	70.8	24.9	32.1	166.8
Valine	242.7	80.5	55.0	470.6	240.5	49.8	147.6	389.9
1-methylhistidine	4.1	1.5	0.9	7.0	5.7*	3.9	1.6	26.4
3-methylhistidine	5.1	5.6	0.5	21.7	6.4	7.5	0.0	39.6
α-aminobutyrate	26.4	9.6	6.6	51.0	25.5	9.1	9.9	52.8
β-aminobutyrate	5.1	1.3	3.0	8.3	5.6	1.3	3.5	8.3
Betaine	52.8	17.8	19.2	82.8	64.8*	20.8	29.4	117.0
β-alanine	6.1	3.7	0.5	13.1	7.1	4.3	1.3	17.6
Citrulline	39.5	15.1	9.7	69.1	40.0	13.5	17.7	81.0
Hydroxyproline	5.9	4.8	1.8	23.3	6.7	3.6	2.1	20.7
Methylnicotinamide	0.232	0.106	0.065	0.550	0.312*	0.166	0.112	0.959
Ornithine	45.6	15.9	8.5	68.6	52.2	16.2	25.8	100.8
Sarcosine	7.5	1.9	3.2	11.0	7.8	2.7	3.4	17.0
Taurine	66.7	29.6	22.8	134.4	60.0	24.0	34.7	156.6
ADMA	0.289	0.100	0.096	0.512	0.344*	0.112	0.101	0.576
L-NAME	0.051	0.015	0.026	0.081	0.056	0.018	0.030	0.102

Values are in μmol/L. *p < 0.05; **p < 0.01.

We have demonstrated many alterations in plasma concentrations of amino acid-related compounds that were dependent on specific disease presentations within the SSc patient group (Table 3). Patients with dcSSc revealed higher concentrations of sarcosine, β-alanine, MNA and N(G)-nitro-L-arginine Methyl Ester (L-NAME) than the lcSSc group. Disease presentation with calcinosis was associated with a significant elevation of glutamate, sarcosine, proline, tyrosine, 3-methylhistidine, and ornithine concentrations. Patients with joint pain have a lower level of plasma glutamine but higher levels of ornithine and 1-methylhistidine. Lung involvement was associated with higher concentrations of valine and arginine. Patients with telangiectasia have a higher concentration of glutamate, lysine, and L-NAME. The presence of extensive skin changes (scleroderma) was associated with lower concentrations of a broad range of AA such as asparagine, sarcosine, proline, histidine, ornithine, citrulline, and phenylalanine.

**TABLE 3:** The concentrations of amino acid metabolites in plasma of systemic sclerosis patients that were significantly different (*p* < 0.05) in patients with specific pattern of disease.

	dcSSc (*n* = 21)				lcSSc (*n* = 21)			
		
	Mean	S.D.	Min	Max	Mean	S.D.	Min	Max
β -alanine	6.05	3.76	1.32	13.3	9.27	4.7	2.83	17.62
Methylnicotinamide	0.31	0.17	0.15	0.95	0.35	0.12	0.22	0.70
Sarcosine	7.35	2.91	3.81	17.03	8.98	2.36	4.22	14.35
L-NAME	0.052	0.012	0.034	0.074	0.065	0.020	0.035	0.102

	**Calcinosis present (*n* = 6)**				**No calcinosis (*n* = 36)**			

Glutamate	146.6	57.5	36.1	205.2	98.3	37.2	41.7	201.5
Proline	246.8	35.9	214.8	315.0	178.5	50.1	102.4	342.6
Tyrosine	99.0	36.1	54.7	148.4	68.9	22.4	42.6	166.7
3-methylhistidine	14.5	13.4	2.3	39.6	5.2	5.7	0.05	22.7
Ornithine	69.3	15.0	44.1	87.7	52.8	15.5	32.6	100.8
Sarcosine	10.1	2.1	8.6	14.3	7.9	2.7	3.8	17.0

	**Joint pain (*n* = 28)**				**No joint pain (*n* = 14)**			

Glutamine	690.6	134.9	457.2	971.1	716.3	121.8	540.8	906.3
Ornithine	58.2	17.1	32.6	100.8	47.5	12.0	33.6	74.5
1-methylhistidine	6.9	1.56	26.3	4.9	4.2	2.5	6.5	1.1

	**Lung involvement (*n* = 26)**				**No lung involvement (*n* = 16)**			

Arginine	81.3	26.4	43.4	167.7	63.7	22.5	38.1	123.9
Valine	249.0	48.6	147.6	346.7	222.5	34.5	178.4	286.6

	**Telangiectasia (*n* = 24)**				**No telangiectasia (*n* = 18)**			

Glutamate	112.7	44.2	36.1	205.2	89.0	39.4	45.2	201.5
Lysine	148.0	30.1	88.3	212.5	130.8	29.9	74.5	203.7
L-NAME	0.07	0.02	0.03	0.10	0.05	0.13	0.03	0.08

	**Scleroderma (*n* = 20)**				**No scleroderma (*n* = 22)**			

Asparagine	34.9	15.2	17.8	83.3	42.0	11.3	20.0	73.0
Proline	167.1	38.7	102.5	235.6	205.0	62.7	100.0	342.7
Histidine	86.6	26.5	49.0	166.0	104.3	26.57	48.9	146.8
Phenylalanine	68.0	24.3	42.0	144.3	85.4	39.6	44.1	207.5
Citrulline	35.7	10.3	17.6	60.7	45.2	16.0	23.3	81.0
Ornithine	49.6	14.4	32.6	77.1	60.8	16.1	44.1	100.8
Sarcosine	7.2	2.6	4.0	13.2	9.2	2.3	5.6	17.0

*Values are in μmol/L.*

## Discussion

In this study, we investigated the plasma amino acid (AA) profile in patients with SSc to detect potential biomarkers and to relate particular changes in the AA pattern with clinical characteristics of patients and organ involvement in the context of potential pathological mechanisms. We indicated a group of AA related compounds that have prevalent fluctuations in this pathology. The most significant changes between SSc patients and healthy controls were observed in plasma 1-methylhistidine (39% increase over control, IOC) > MNA (35% IOC) > betaine (23% IOC) > tryptophan (20% decrease over control, DOC) > ADMA (19% IOC) > proline (17% IOC) > glutamine (12% IOC). Moreover, we have demonstrated significant alterations in the plasma AA levels within the SSc patient group that deserve attention as potential molecules that may predict the specific course of the disease and are worth further studies as clinical biomarkers. The disturbed balance of the AA metabolites that we observed is known to be associated with vascular endothelial dysfunction, inflammation, or methylation disturbances.

Cardiovascular dysfunction is of fundamental importance in SSc pathogenesis from the early onset of the disease through the late clinical complications (Silva et al., 2015; Smoleńska et al., 2019). In this study, we revealed an increased plasma concentration of the endogenous NO synthase (NOS) inhibitor – ADMA, that was increased in the entire SSc patients’ group in comparison to healthy controls. The impaired nitric oxide production leads to the augmentation of vasoconstrictor episodes and pathological changes in the vascular system such as inflammatory and thrombotic signaling or vascular remodeling (Flavahan, 2015). Moreover patients with SSc expressed a decreased level of plasma tryptophan. This AA could be utilized by endothelial cells and via the kynurenine pathway may direct them to ROS-dependent apoptosis (Duran and San Martín, 2014). Interestingly, patients with specific clinical presentations such as dcSSc and telangiectasia revealed an elevated level of other NOS inhibitor - L-NAME, suggesting increased severity of the endothelial dysfunction that could mark active disease pattern and more extended vascular injury. Therefore, our research highlights the importance of multi-parameter profiles of endothelium analysis in patients with SSc that will help to track the severity of endothelial dysfunction and contribute to the monitoring of endothelium protection therapy.

The pathogenesis of SSc involves the Th1-related early inflammatory phase, which is then followed by a switch to Th2, leading to irreversible fibrosis. However, in some subgroups of SSc patients, there is a prolonged active inflammatory response. Therefore, specific inflammatory markers were proposed as important candidates for the diagnosis and differentiation of the form of the disease (Ross et al., 2018). Overall, patients with inflammatory SSc seems to represent a subgroup with higher morbidity and mortality. Accordingly, patients with inflammatory SSc showed a faster decline of forced vital capacity (FVC) over time and received more frequently immunosuppressive treatment (e.g., cyclophosphamide, CYC) (Mitev et al., 2019). It has been demonstrated that these patients showed persistent long-term CRP elevations and even treatment with CYC did not affect CRP levels. Presumably, macrophages, less affected by CYC treatment, maintain inflammation in this subgroup. In our patient group, systemic inflammation may be responsible for the increased concentration of MNA in dcSSc, while elevated sarcosine and β-alanine may relate to more severe pathological processes and muscle injury. Moreover, the consistent increase of betaine, 1-methylhistidine, and MNA (by 20–40%) in SSc patients in comparison to healthy controls implies the activation of methylated derivatives formation. One challenge is that these compounds are produced by completely different methylation mechanisms: methylation of histidine residues in skeletal muscle proteins in the case of 1-methyhistidine, methylation of free nicotinamide by liver-specific enzyme in the case of MNA, and methylation of phospholipid elements in the case of betaine (Houweling et al., 2012; Obeid, 2013; Pissios, 2017). One common factor that is needed in all these processes is S-adenosylmethionine. However, it needs to be established whether the production or turnover of this molecule is indeed stimulated in patients with SSc.

The disease presentation with calcinosis was associated with a significant elevation of glutamate, sarcosine, proline, tyrosine, 3-methylhistidine, and ornithine concentration. It is difficult to speculate on the mechanism but the broad range and unidirectional trend for amino acid changes may indicate its reduced utilization in general that may be related to impaired mobility. Patients with joint pain have a lower level of plasma glutamine but higher of ornithine and 1-methylhistidine. That again may relate to impaired mobility or in the case of glutamine to impaired immune system function (Cruzat et al., 2018).

The presence of extensive skin changes (scleroderma) was associated with lower concentrations of asparagine, sarcosine, proline, histidine, ornithine, citrulline, and phenylalanine. Such a broad range of changes may indicate accelerated protein synthesis associated with this specific disease presentation. In turn, proline deficiency, which is an essential amino acid during periods of increased body stress, in patients with scleroderma can be an indicator of poor prognosis (Liang et al., 2013). Extensive skin involvement corresponds to more severe internal organ manifestation and increased impairment (Wu, 2009). The differences in intestinal absorption or diet may also contribute. Histidine and phenylalanine are nutritionally essential amino acids and their lower levels suggest that there are disturbances in absorption from the digestive tract in scleroderma patients. Even 90% of cases with SSc involve the digestive tract suggesting the need for appropriate nutritional care, treatment, and dedicated monitoring (Miller et al., 2018).

One compound that differentiated several groups was sarcosine. This molecule is an intermediate of glycine metabolism, being a specific substrate of sarcosine dehydrogenase that is a mitochondrial enzyme, which converts sarcosine to glycine. Sarcosine is formed from betaine via dimethylglycine by dimethylglycine dehydrogenase (Augustin et al., 2016). Concentration of sarcosine was lower in dcSSc patients and in those with scleroderma while higher concentration was observed in calcinosis presentation. No differences in betaine concentration indicate that its conversion to glycine is responsible for the changes in sarcosine concentration. While the exact mechanism of differences in sarcosine concentration is difficult to propose at present, this molecule is worth further investigation as a biomarker of specific disease complications.

## Conclusion

This study confirmed that the pathobiology of systemic sclerosis interferes with the plasma amino acid profile and particular amino acid metabolites can be selected as potential biomarkers and predictive factors that differentiate individual subtypes of systemic sclerosis.

## Data Availability Statement

All datasets presented in this study are included in the article/supplementary material.

## Ethics Statement

The studies involving human participants were reviewed and approved by Ethics Committee at the Medical University of Gdańsk. The patients/participants provided their written informed consent to participate in this study.

## Author Contributions

ZS conceived the study, conducted data analysis, and wrote the manuscript. MZ-K conducted biochemical analysis. AW conducted clinical data analysis. BK-Z wrote the manuscript. ZZ corrected the manuscript. All authors contributed to the article and approved the submitted version.

## Conflict of Interest

The authors declare that the research was conducted in the absence of any commercial or financial relationships that could be construed as a potential conflict of interest.
